# Patterns of gene expression and DNA methylation in human fetal and adult liver

**DOI:** 10.1186/s12864-015-2066-3

**Published:** 2015-11-21

**Authors:** Susan M. Huse, Philip A. Gruppuso, Kim Boekelheide, Jennifer A. Sanders

**Affiliations:** Department of Pathology and Laboratory Medicine, Brown University, Providence, RI USA; Department of Pediatrics, Rhode Island Hospital and Brown University, Providence, RI USA; Department of Molecular Biology, Cell Biology and Biochemistry, Brown University, Providence, RI USA

**Keywords:** Gene expression, Methylation, Liver, Fetal Development

## Abstract

**Background:**

DNA methylation is an important epigenetic control mechanism that has been shown to be associated with gene silencing through the course of development, maturation and aging. However, only limited data are available regarding the relationship between methylation and gene expression in human development.

**Results:**

We analyzed the methylome and transcriptome of three human fetal liver samples (gestational age 20–22 weeks) and three adult human liver samples. Genes whose expression differed between fetal and adult numbered 7,673. Adult overexpression was associated with metabolic pathways and, in particular, cytochrome P450 enzymes while fetal overexpression reflected enrichment for DNA replication and repair. Analysis for DNA methylation using the Illumina Infinium 450 K HumanMethylation BeadChip showed that 42 % of the quality filtered 426,154 methylation sites differed significantly between adult and fetal tissue (*q* ≤ 0.05). Differences were small; 69 % of the significant sites differed in their mean methylation beta value by ≤0.2. There was a trend among all sites toward higher methylation in the adult samples with the most frequent difference in beta being 0.1. Characterization of the relationship between methylation and expression revealed a clear difference between fetus and adult. Methylation of genes overexpressed in fetal liver showed the same pattern as seen for genes that were similarly expressed in fetal and adult liver. In contrast, adult overexpressed genes showed fetal hypermethylation that differed from the similarly expressed genes. An examination of gene region-specific methylation showed that sites proximal to the transcription start site or within the first exon with a significant fetal-adult difference in beta (>0.2) showed an inverse relationship with gene expression.

**Conclusions:**

Nearly half of the CpGs in human liver show a significant difference in methylation comparing fetal and adult samples. Sites proximal to the transcription start site or within the first exon that show a transition from hypermethylation in the fetus to hypomethylation or intermediate methylation in the adult are associated with inverse changes in gene expression. In contrast, increases in methylation going from fetal to adult are not associated with fetal-to-adult decreased expression. These findings indicate fundamentally different roles for and/or regulation of DNA methylation in human fetal and adult liver.

## Background

Epigenetic regulation of gene expression allows for the transmission of heritable traits and environmental effects on phenotype in a manner independent of the DNA sequence [1]. DNA methylation has been identified as an important epigenetic control mechanism, particularly in large-scale gene silencing, such as X-inactivation [2], genomic imprinting [3], and silencing of transposable elements in plants [4, 5]. Gene silencing associated with DNA methylation has been found to occur through the course of development, maturation and aging [6, 7].

DNA methylation, most often involving the addition of a methyl group to a cytosine in the context of a CpG site, can occur actively or passively. Passive demethylation is the loss of methylation marks during DNA replication [8], whereas active demethylation is the specific removal of methylation marks. Genome-wide demethylation, an event thought to occur during early development, is followed by remethylation of imprinted genes and genes encoded on the X-chromosome. DNA methylation that is essential for embryonic cell differentiation is erased during fertilization. Gene silencing methylation then progressively accumulates as stem cells irreversibly differentiate into various cell types [9]. Gene-specific demethylation can also occur in response to environmental stress [8, 10] or in association with gene upregulation as a mechanism of drug resistance [11].

DNA methylation occurs throughout the genome in complex patterns and in varying gene regions, including promoter regions. In general, DNA methylation of promoter regions is correlated with reduced expression. However, some methylation patterns within the gene body are paradoxically associated with increased gene expression [12, 13].

While there is an established role for DNA methylation in the epigenetic regulation of gene expression, only limited data have been published regarding this relationship in human development. Our laboratory has a long-standing interest in the regulation of fetal liver development and programming of liver metabolism [14–18]. Elucidating the mechanisms involved in the developmental programming of hepatocyte growth, differentiation and metabolism is critical to understanding the fetal origins of Type 2 diabetes and metabolic syndrome [19, 20]. In this study, we compare DNA methylation and gene expression from liver tissue of human fetal and adult donors to examine the regulatory role of this epigenetic mechanism in the context of normal liver developmental physiology. We used the Illumina Infinium 450 K HumanMethylation BeadChip to assess methylation rates at over 480,000 CpG locations across the human genome, and the Affymetrix Human Gene ST 1.0 array to measure gene expression for 33,297 genes. Our findings point to complex and differing functional regulation of gene expression by DNA methylation in mid-gestation fetal and adult humans.

## Results

### Characterization of the fetal and adult liver transcriptome and DNA methylome

We analyzed triplicate human fetal and adult liver samples. All six samples were derived from males to avoid differences related to sex-associated imprinting. To minimize the variance due to developmental stage or age, we selected samples from a narrow window: spontaneous fetal losses that occurred between 20 and 22 weeks gestation, and normal adult liver from individuals who were 55 to 62 year olds. Histological analysis of the fetal liver using hematoxylin and eosin-stained sections showed normal architecture with no evidence of tissue injury or necrosis.

**Fig. 1:**
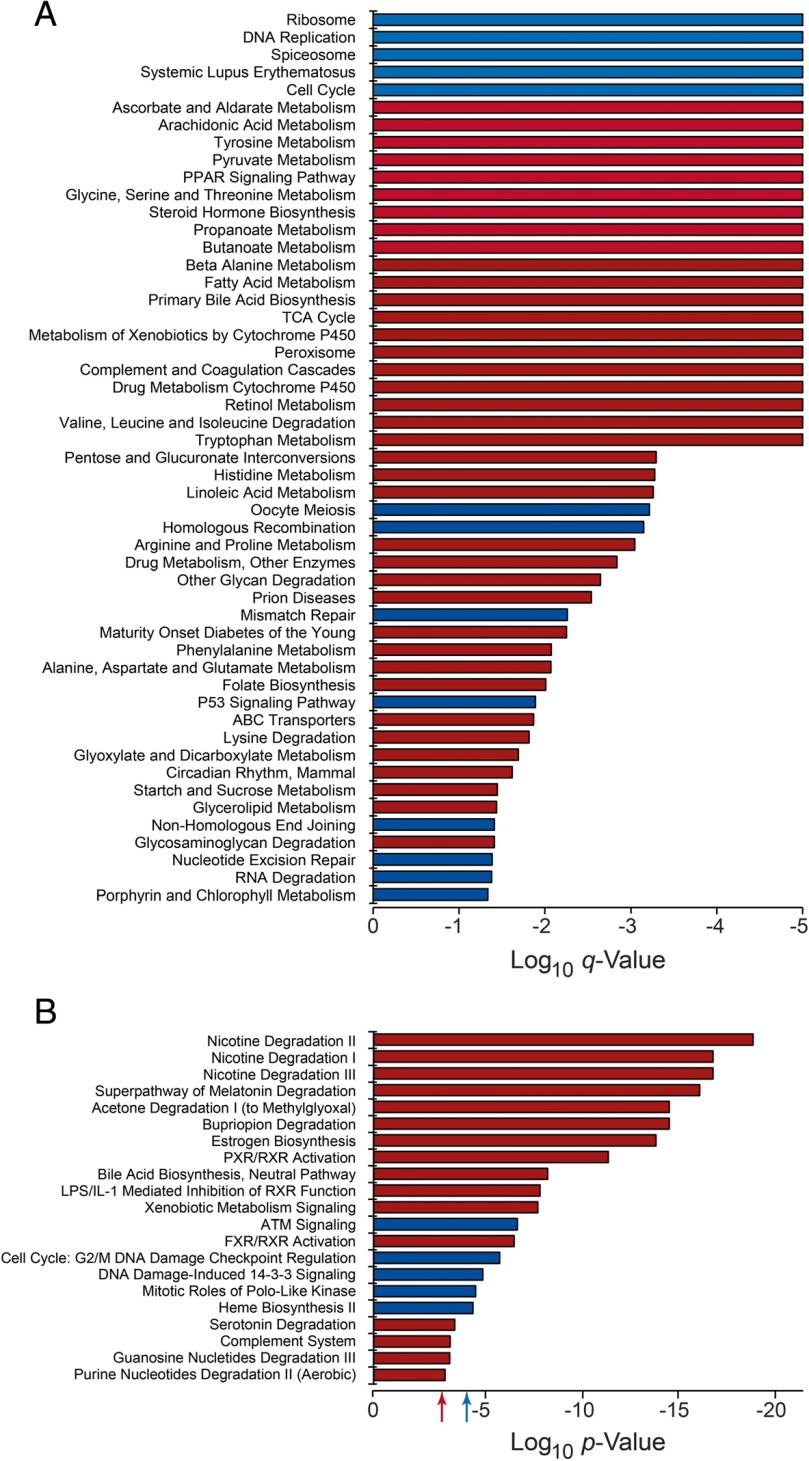
Analysis of gene expression in fetal and adult liver. Microarray data were analyzed by GSEA (**a**) and IPA (**b**). *Blue bars* and *red bars* reflect higher expression in the fetal and adult samples, respectively. Results are stratified based on level of significance. GSEA data are shown as the FDR *q*-value. IPA results show the unadjusted *p*-value. The threshold for significance of the IPA results was determined using fetal (*blue arrow*) and adult (*red arrow*) control data sets, as described in Methods

To characterize the pattern of differential gene expression in the fetal and adult liver samples, we used Gene Set Enrichment Analysis (GSEA; Fig. 1a) [21]. A number of gene sets, including Ribosome, DNA Replication, Systemic Lupus Erythematosus, Cell Cycle, and Mismatch Repair, were enriched in fetal liver. Enrichment of these gene sets reflected changes in the expression of cell cycle constituents, histones, DNA replication factors and genes involved in RNA processing. Conversely, many of the gene sets enriched in adult liver reflected greater functional differentiation. Among these were several gene sets that were accounted for by higher expression of cytochrome P450 enzymes, transporters and complement factors. A number of other gene sets enriched in adult liver were accounted for by genes encoding enzymes involved in lipid and amino acid metabolism.

We identified 7,673 genes whose expression level differed significantly between the fetal and adult liver samples (False Discovery Rate [FDR] *q*-value ≤0.05). We based a priori gene selection for Ingenuity Pathway Analysis (IPA®) on a significant difference in mean expression between the two groups of 5-fold. This resulted in 594 input genes. Of these genes, 384 were overexpressed in fetal liver and 210 were overexpressed in adult liver. IPA results (Fig. 1b) were largely accounted for by higher expression of genes associated with DNA replication in fetal liver and cytochrome P450 enzymes in adult liver.

DNA methylation array analysis yielded 426,154 methylation locations along the human genome that passed quality filtering. We found that 179,284 of these sites (42 %) differed significantly between adult and fetal tissue (*q* ≤ 0.05). Despite statistically significant differences, we found that the majority of these CpG sites did not differ greatly in their overall level of methylation; 69 % of the statistically significant sites (124,392) showed a difference in their mean methylation beta value (delta beta; dβ) of less than 0.2. Only 5 % of the statistically significant sites (8,151) showed a mean dβ greater than 0.5.

The consistency of methylation within groups was high. The mean variance for all fetal methylation sites was 0.0011; the mean variance for the adult samples was 0.0017. Of all CpGs in both groups, 96 % had a variance <0.01. Thus, the variance within groups was much less than the change in methylation between groups despite the fact that mean differences were generally small.

**Fig. 2:**
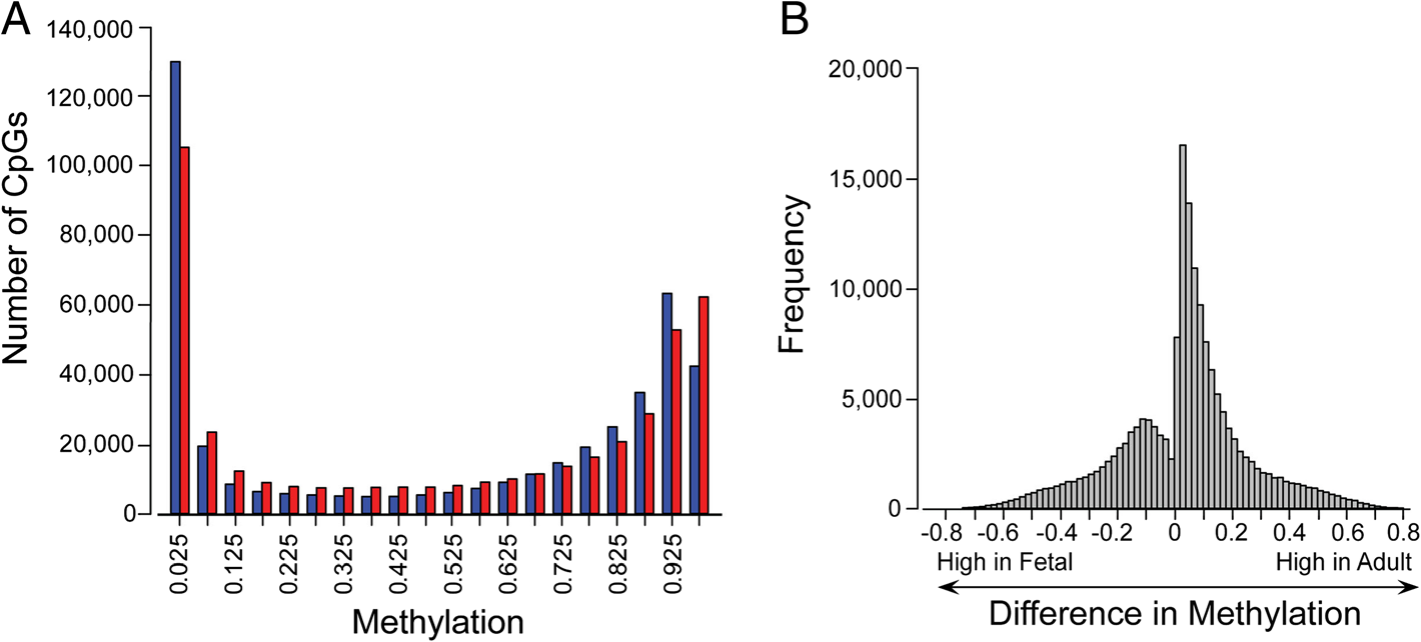
Distribution of methylation data. Methylation sites were stratified based on the average methylation level. **a** A histogram showing the number of CpGs at any given methylation level was generated using intervals of 0.05 for the range of 0 to 1. All methylation sites were included in the determination of the mean of triplicate samples for each site. Fetal results are shown in *blue* and adult in *red*. **b** The difference in methylation between fetal and adult data sets, calculated as the average delta beta for each site, is shown as a histogram. For this analysis, sites were included only if there was a *q*-significant difference between fetal and adult

Of the sites that differed significantly between fetal and adult, but for which dβ was ≤0.2, 72 % of sites showed higher methylation in the adult samples. The significantly different sites (30,495) with dβ >0.2 were also more likely to be hypermethylated in the adult samples (56 %). Of the markedly undermethylated CpGs (beta <0.3), most of the fetal CpGs (74 %) had a mean dβ <0.05 in contrast to only 36 % of the corresponding adult CpGs. Graphic representation (Fig. 2a) showed that the differences between fetus and adult across the full methylation range were subtle. The dβ distribution (Fig. 2b) showed a slight shift toward higher methylation in the adult samples with the most frequent delta beta approximating 0.01.

### Distribution of DNA methylation within gene regions

The gene region in which DNA methylation occurs contributes to the complexity of its effects on transcription [22]. We looked at two annotation sets to classify methylation effect by gene region, regulatory feature group (including “promoter associated”) and reference gene group (including “upstream of transcription start site”). Unfortunately, 79 % of the CpGs did not have regulatory feature group annotations, whereas 75 % of the CpGs did have the reference gene group. A preliminary analysis of the 8,151 statistically different sites showing a dβ > 0.5 described above, show that only 14 % had a regulatory feature group annotation, of which 92 % (1,049) were specifically “promoter associated”. These promoter-associated sites fell on 508 genes, more than 80 % of which (416) did not show a significant change in expression (q ≤ 0.05, fold change > 2). Given the low levels of annotation for regulatory feature group, and the ambiguous findings they provided, we chose instead to use the reference gene group annotations.

**Fig. 3:**
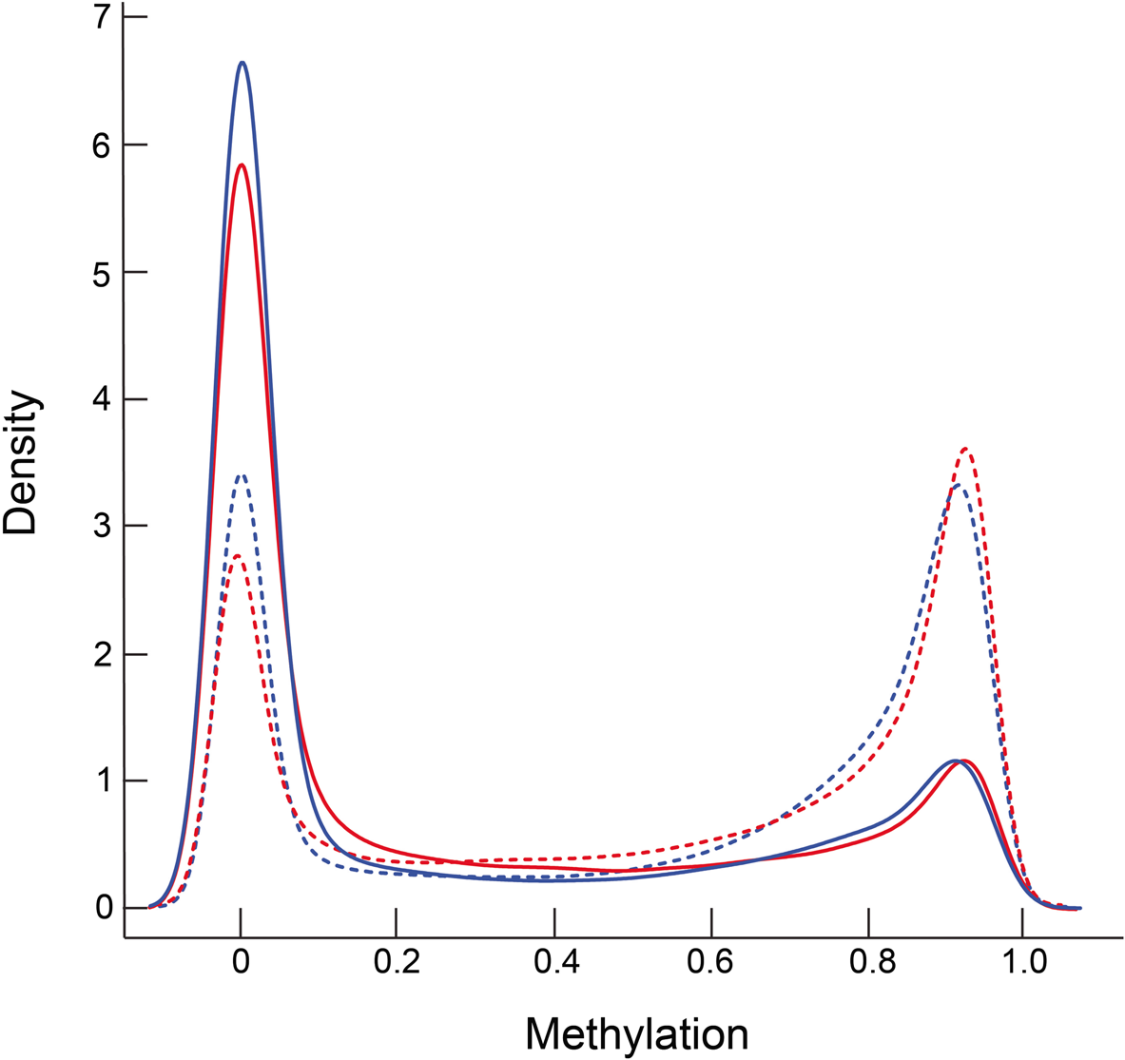
Distribution of DNA methylation based on gene region. Triplicate fetal (*blue*) and adult (*red*) samples were analyzed using all CpGs that were found to be methylated. The *solid lines* represent sites within 1,500 base pairs of the transcription start site or within the first exon, and the *dashed lines* those sites that are within the gene body, 5’UTR or 3’UTR

We characterized CpG methylation reference gene groups into two distinct regions. Sites within 200 or 1,500 base pairs of the transcription start site (TSS200 and TSS1500, respectively) or within the first exon were aggregated and designated as TSS/Ex1. The remaining regions, 5’UTR, 3’UTR and gene body, were aggregated and designated as UTR/GB [23, 24]. This approach to combining sites was based on the observation that DNA methylation of the promoter regions upstream of the transcription start site has been linked to reduced expression while the methylation along the gene body can have the converse effect [22, 13]. In addition, Brenet et al. [25] describe the 1^st^ exon as being tightly linked to transcription. The appropriateness of this approach was indeed confirmed by subsequent analyses (*vide infra*). Using this approach, we found that the TSS/Ex sites were much more likely to be hypomethylated than hypermethylated (Fig. 3). This was in contrast to the UTR/GB sites, which showed similar proportions of hypo- and hypermethylation. This pattern of site-related methylation was similar between the fetal and adult samples.

**Fig. 4:**
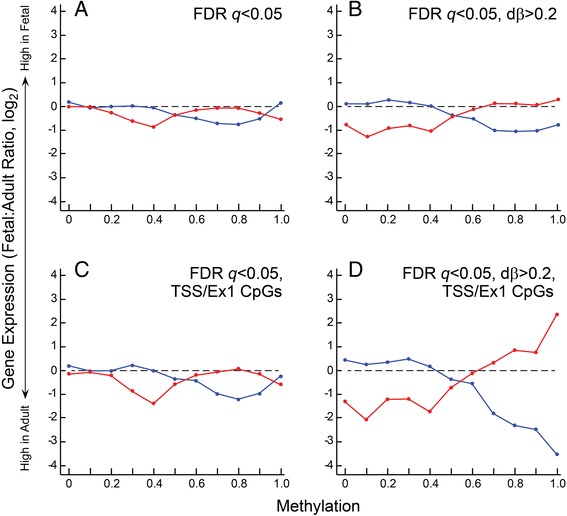
The relationship between DNA methylation and gene expression. Results are shown as the average methylation for each gene (mean beta value for the sites on that gene) versus the mean fetal:adult expression ratio for each gene. Data for the three fetal samples (*blue*) and the three adult samples (*red*) were filtered using various parameters to generate the input for the analysis. **a** All genes for which methylation was significantly different for fetal versus adult (*q* < 0.05). **b** Genes for which the fetal:adult difference in methylation (dβ) was at least 0.2. **c** Significant methylation; methylation restricted to TSS/Ex1 CpGs. **d** Significant methylation; methylation restricted to TSS/Ex1 CpGs for which dβ was at least 0.2

We went on to examine the relationship between site of methylation and gene expression. To do so, we created a hierarchy for selection of methylation sites. We found little if any relationship between methylation and gene expression when all statisticallt significant methylation events were included in the analysis (Fig. 4a). Additional filtering based on dβ >0.2 (Fig. 4b) had little effect. The same was true when we limited our analysis to TSS/Ex1sites (Fig. 4c). However, combined filtering for dβ >0.2 and TSS/Ex1 location showed a strong relationship (Fig. 4d). Genes for which TSS/Ex1 methylation was high in the fetus were relatively overexpressed in adult liver. Conversely, genes with high TSS/Ex1 methylation in adult liver were relatively overexpressed in fetal liver. Low and intermediate levels of TSS/Ex1 methylation did not appear to be correlated with relative expression levels in fetal liver. Low methylation in adult liver was associated with relatively higher expression in adult liver, though this relationship was not as apparent as was the case for hypermethylated TSS/Ex1 CpGs. Combined filtering for dβ >0.2 and UTR/GB location (data not shown) yielded results similar to those seen when we filtered for significant methylation events and dβ >0.2 (Fig. 4b).

### The relationship between methylation and gene expression in fetal and adult liver

**Fig. 5:**
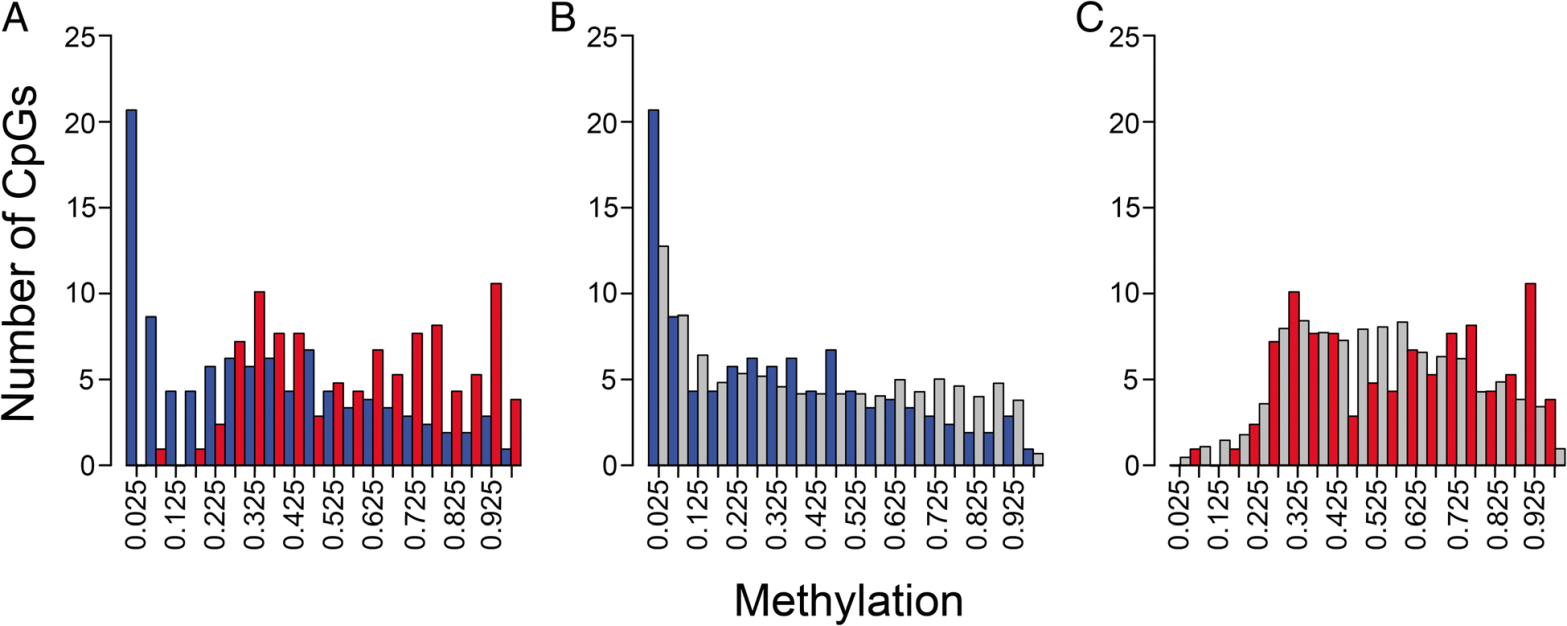
Methylation of genes overexpressed in fetal liver. Overexpression was defined as a *q*-significant 5-fold difference in mean expression level between fetal and adult. **a** Fetal (*blue*) and adult (*red*) TSS/Ex1 methylation (FDR *q* < 0.05 with dβ >0.2) results are shown juxtaposed against one another. Also shown are methylation results for fetal (**b**) and adult (**c**) liver relative to data generated using randomly selected gene sets for which fetal and adult liver expression was similar (fetal:adult ratio of −1.1 to +1.1; gray bars)

To explore the potential role of TSS/Ex1 methylation in regulating gene expression, we examined the methylation status of this region of genes that were overexpressed in fetal or adult liver as defined by a significant (*q* ≤ 0.05) fold-difference of at least 5. For comparison purposes, we expanded our analysis to include randomly selected genes for which expression was similar in fetal and adult liver. For this comparison group, we selected 4,631 genes represented on both the Affymetrix and the Illumina Infinium arrays for which the fold-difference in expression between fetal and adult liver was non-significant and less than 1.1. A comparison of fetal and adult TSS/Ex1 methylation for genes overexpressed in fetal liver showed what appeared to be a fetal versus adult difference in methylation (Fig. 5a). Fetal liver showed hypomethylation of these genes while adult liver showed intermediate methylation and hypermethylation. However, these methylation patterns in both fetal liver (Fig. 5b) and adult liver (Fig. 5c) were similar to those seen for the control data set (genes with similar fetal and adult expression).

**Figure 6:**
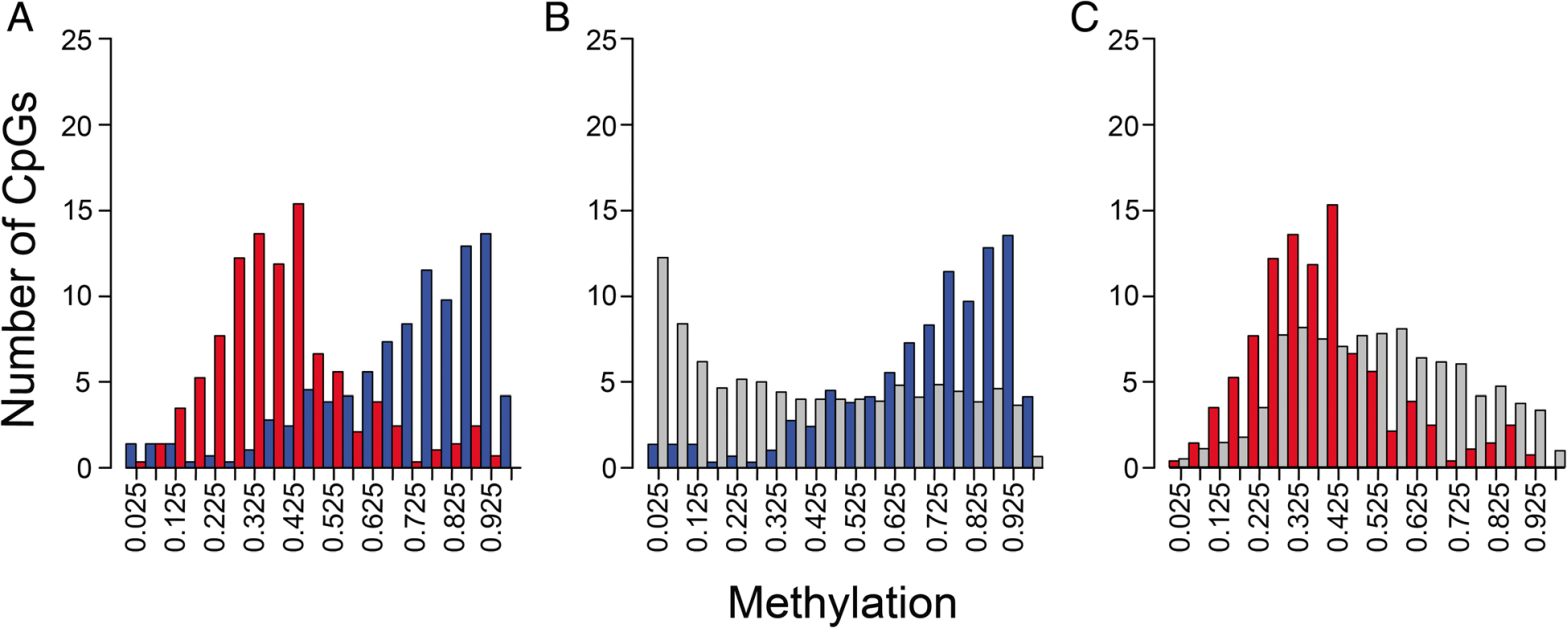
Methylation of genes overexpressed in adult liver. Overexpression was defined as a *q*-significant 5-fold difference in mean expression level between fetal and adult. **a** Fetal (*blue*) and adult (*red*) TSS/Ex1 methylation (FDR *q* < 0.05 with dβ >0.2) results shown juxtaposed against one another. Also shown are methylation results for fetal (**b**) and adult (**c**) liver relative to data generated using randomly selected gene sets for which fetal and adult liver expression was similar (fetal:adult ratio of −1.1 to +1.1; gray bars)

In contrast, genes overexpressed in adult liver had TSS/Ex1 methylation patterns in fetal and adult liver that not only differed from one another (Fig. 6a), but also differed from the equally expressed genes. TSS/Ex1 methylation in fetal liver was high relative to the control data set (Fig. 6b). In adult liver, methylation was low to intermediate (Fig. 6c). Histograms based on the promoter-associated regulatory feature group display similar patterns, but have insufficient CpGs to be of statistical significance (data not shown). Of note, there were very few unmethylated sites in adult tissue from any of the three expression data sets.

We hypothesized that genes for which methylation differed significantly between fetal and adult liver would represent particular functional categories. To test this hypothesis, we selected genes that were overexpressed in fetal or adult liver as defined by a significant (*q* ≤ 0.05) fold-difference of at least 5, and that showed a methylation difference (dβ >0.2, *q* ≤ 0.05) in the TSS/Ex1 region. These 189 genes were entered into IPA. As a control group to assess level of significance, we also analyzed five equally sized sets of genes for which the fetal:adult expression ratio was between −1.1 and 1.1. Results (Table 1) show pathways involved in drug and xenobiotic metabolism were significant. These pathways represent a large subset of the categories found to be significant in adult liver (Fig. 1b). They were largely accounted for by genes encoding cytochrome p450 enzymes.

**Table 1:** I PA categories, genes and p-values for Cannonical Pathways

Pathway	Genes	P-value
Nicotine Degradation II	FMO3,CYP1A1,UGT2B7,CYP2C18,CYP2C9,INMT,CYP1B1,UGT2B15, CYP1A2,CYP2D6,CYP2E1,CYP2A6,CYP2B6,AOX1,CYP2C8	5.62 × 10^−18^
Nicotine Degradation III	CYP1A1,UGT2B7,CYP2C18,CYP2C9,CYP1B1,UGT2B15,CYP1A2,CYP2E1, CYP2D6,CYP2A6,AOX1,CYP2B6,CYP2C8	1.54 × 10^−15^
Bupropion Degradation	CYP1A1,CYP1A2,CYP2E1,CYP2D6,CYP2C18,CYP2C9,CYP2A6,CYP2B6, CYP1B1,CYP2C8	5.92 × 10^−14^
Acetone Degradation I (to Methylglyoxal)	CYP1A1,CYP1A2,CYP2E1,CYP2D6,CYP2C18,CYP2C9,CYP2A6,CYP2B6, CYP1B1,CYP2C8	5.92 × 10^−14^
Melatonin Degradation I	CYP1A1,CYP1A2,UGT2B7,CYP2E1,CYP2D6,CYP2C18,CYP2C9,CYP2A6, CYP2B6,CYP1B1,CYP2C8,UGT2B15	6.05 × 10^−14^
Estrogen Biosynthesis	CYP1A1,CYP1A2,CYP2E1,CYP2D6,CYP2C18,CYP2C9,CYP2A6,CYP2B6, AKR1C4,CYP1B1,CYP2C8	8.47 × 10^−14^
Superpathway of Melatonin Degradation	CYP1A1,CYP1A2,UGT2B7,CYP2E1,CYP2D6,CYP2C18,CYP2C9,CYP2A6, CYP2B6,CYP1B1,CYP2C8,UGT2B15	1.61 × 10^−13^
PXR/RXR Activation	CYP1A2,CES3,CYP2C9,CYP2A6,CYP2B6,SULT2A1,CYP2C8	7.87 × 10^−6^
LPS/IL-1 Mediated Inhibition of RXR Function	FMO3,SLC27A5,SLC10A1,ACSL4,CYP2C9,CYP2A6,CYP2B6,ABCC4, SULT2A1,CYP2C8	3.92 × 10^−5^

In addition to the previous analysis, we examined the region-specific methylation data using a categorical approach. We defined hypomethylation, intermediate methylation and hypermethylation as beta ranges from 0 to 0.3, 0.3 to 0.7 and 0.7 to 1.0, respectively. We again defined location as TSS/Ex1 or UTR/GB. Genes were defined as fetal overexpressed or adult overexpressed based on 5-fold differences. Similarly expressed genes were defined as those with a mean fetal:adult expression ratio of -1.1 to 1.1. The Chi-Square test was used to assess statistical significance. Results (Table 2) showed that both fetal overexpressed and adult overexpressed genes differed significantly from similarly expressed genes with regard to fetal and adult TSS/Ex1 methylation. However, the fetal and adult methylation patterns were similar. Therefore, we cannot assign biological significance to these differences. Fetal and adult methylation of UTR/GB sites was similar comparing both fetal and adult overexpressed genes with similarly expressed genes. We interpreted this analysis as indicating that TSS/Ex1 hypomethylation was more common for genes that showed significantly different fetal versus adult expression than for genes whose expression was similar in fetal and adult liver. However, hypomethylation of these regions did not correlate functionally with the changes in gene expression as fetal overexpressed genes show a similar level of upstream hypomethylation in both the fetus and adult.

**Table 2:** Percentage of CpG sites by location category and methylation level

Gene Expression	Tissue	Location on Gene	Number of CpGs	Hypomethylation (%)	Intermediate Methylation (%)	Hypermethylation (%)
Fetal Overexpressed	Fetal	TSS/Ex1	1640	76	10	14
		UTR/GB	2312	31	11	58
	Adult	TSS/Ex1	1640	71	11	18
		UTR/GB	2312	27	15	58
Adult Overexpressed	Fetal	TSS/Ex1	912	47	16	37
		UTR/GB	957	24	16	61
	Adult	TSS/Ex1	912	56	24	20
		UTR/GB	957	24	21	55
Similarly Expressed	Fetal	TSS/Ex1	2464	43	34	23
		UTR/GB	4983	31	37	32
	Adult	TSS/Ex1	2464	16	60	23
		UTR/GB	4983	12	57	30

CpGs were selected based on their relationship to genes whose expression differed significantly between fetal and adult liver (*q* < 0.05; fold-difference >5), or their relationship to genes that were similarly expressed in the two groups (fold-difference ≤1.1). Based on criteria described in the text, CpGs were then categorized as localized to within 200 or 1,500 base pairs of the transcription start site or the first exon (TSS/Ex1), or within the 5’UTR, 3’UTR or gene body, and as showing hypo-, intermediate or hypermethylation

To understand the utility of using DNA methylation changes to predict gene expression, we calculated the probability that a significant change in methylation of any CpG would be associated with a difference in the fetal:adult expression ratio (Table 3). CpGs that showed hypermethylation in the fetal samples relative to adult were associated with fetal underexpression of the associated genes. TSS/Ex1 CpGs with a fetal-to-adult methylation decrease of 0.5 or more was the best predictor; 33 % of these CpGs were associated with adult overexpression with a fold change of at least 2. TSS/Ex1 CpGs with a fetal-to-adult methylation decrease of 0.2 or more had a 20 % probability of association with 2-fold or greater overexpression in the adult samples. UTR/GB CpGs were about half as predictive as TSS/Ex1 CpGs. In contrast, neither an increase in fetal to adult of dβ >0.2 nor even the more stringent dβ >0.5 was associated with relative adult underexpression regardless of location category.

**Table 3:** Prediction Probability of DNA Methylation Change to Gene Expression Change

			Gene expression induced in adult	
Fetal Hypermethylation	Location	Number of CpGs	>2-Fold	>5-Fold
Fetal-to-Adult Decrease >0.2	TSS/Ex1	3,510	20 %	7 %
	UTR/GB	8,807	12 %	2 %
Fetal-to-Adult Decrease >0.5	TSS/Ex1	391	33 %	11 %
	UTR/GB	1,350	14 %	2 %
			Gene Expression Induced in Fetus	
Adult Hypermethylation	Location	Number of CpGs	>2-Fold	>5-Fold
Fetal-to-Adult Increase >0.2	TSS/Ex1	22,587	7 %	2 %
	UTR/GB	54,769	5 %	2 %
Fetal-to-Adult Increase >0.5	TSS/Ex1	25,706	7 %	2 %
	UTR/GB	62,226	5 %	2 %

CpGs were categorized based on fetal-to-adult differences in methylation status and their location within genes (TSS/Ex1 versus UTR/GB). Their predictive value was assigned based on the fetal:adult difference in expression of the genes to which the respective CpGs were assigned

**Fig. 7:**
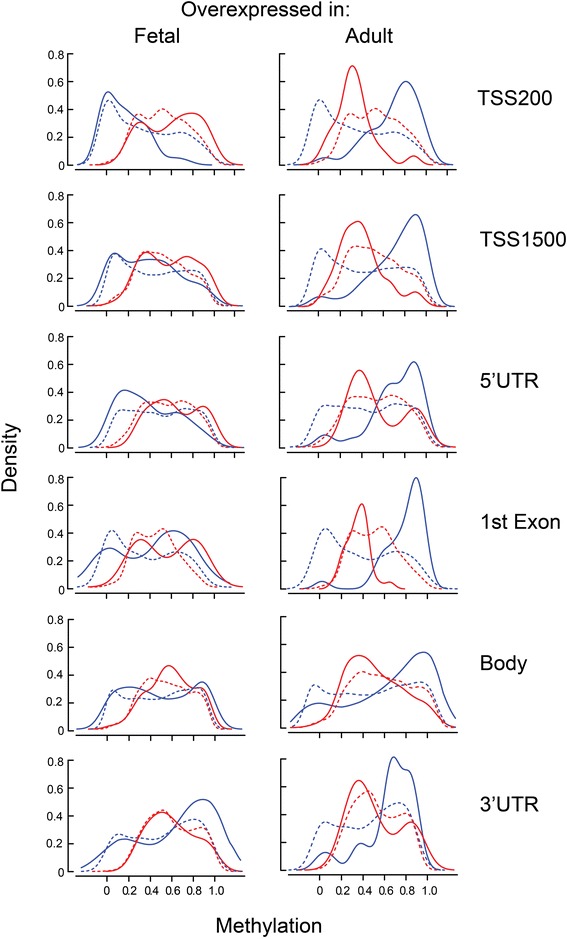
Distribution of gene region-specific methylation for genes overexpressed in fetal or adult liver. For all graphs, the density of fetal methylation is shown in blue and adult in red. Solid lines represent the density plots for genes that are 5-fold overexpressed in fetal liver (*left*) or adult liver (*right*). Results for genes that were equally expressed in fetal and adult liver (fetal:adult ratio of −1.1 to +1.1) are shown for comparison purposes as dotted lines. Methylation data were filtered based on a significant (*q* < 0.05) difference of at least 0.2. The graphs are grouped in rows based on gene regions: CpGs within 200 base pairs of the transcription start site (TSS200), within 1,500 base pairs of the transcription start site (TSS1500), or within the 5’UTR, 1st exon, gene body, or 3’UTR

To more thoroughly characterize the relationship between gene region-specific methylation and gene expression, we performed an analysis in which results were separated based on CpG position within the various gene regions – TSS200, TSS1500, 5’UTR, 1^st^ exon, gene body, or 3’UTR. The analysis was restricted to methylation events that differed significantly between fetal and adult liver (FDR *q* < 0.05) with dβ >0.2. Results (Fig. 7) showed a clear distinction between fetal overexpressed and adult overexpressed genes. The former showed little to no difference at any methylation site when compared to equally expressed genes. In contrast, adult overexpressed genes showed hypermethylation in fetal liver and intermediate methylation in adult liver relative to equally expressed genes. Hypermethylation in fetal liver appeared to be consistent across both region categories. Intermediate methylation exclusive of hypermethylation in the adult was confined to the TSS locations.

## Discussion

The goal of the present study was to characterize patterns of DNA methylation in human fetal and adult liver tissue so as to examine their relationship to patterns of gene expression. We chose a direct comparison of liver tissue from mid-gestation human fetuses and adult male subjects for several reasons. While certainly not homogeneous, the majority of RNA and DNA from an adult human liver sample is derived from hepatocytes, which make up about 60 % of the cells and about 80 % of the liver mass [26]. Although the fetal liver samples were obtained from mid-gestation spontaneous miscarriages, the expression and methylation results we obtained showed minimal variance between samples. Variance among adult samples was similarly low. We interpreted this as indicating the absence of confounding secondary effects of the tissue donors’ condition on our analyses.

Liver tissue contains a mix of cell types that may have different patterns of DNA methylation, which can affect the average liver tissue methylation patterns. In addition, functional heterogeneity among individual cell types, including hepatocytes [27], may contribute to cell-to-cell variations in methylation. Fetal liver presents another potential challenge in that it is a hematopoietic organ; however, hematopoietic genes were not particularly prominent among the genes detected in our expression arrays.

Notwithstanding these potential limitations, we have made several observations that are supported by multiple analyses. The first is that nearly half of the detected methylation sites differed significantly between fetal and adult liver. With regard to the relationship between methylation and gene expression, our data indicate that there may be fundamentally different regulatory roles in fetal and adult liver. Genes that were overexpressed in the fetus did not differ in their methylation pattern from genes that showed similar expression levels in the fetal and adult samples. In contrast, genes that were overexpressed in the adult showed the expected inverse relationship to methylation. This was especially apparent among the sites upstream from the TSS or within the first exon and that showed relatively greater differences in methylation between the fetal and adult samples.

Although our study looked at only two points in time, our findings are consistent with so-called “epigenetic drift” in which DNA methylation patterns diverge over time, either through stochastic changes in methylation or through responses to differing environmental conditions [28–30]. We found that fetal versus adult differences in methylation tended to be small; about 80 % of significantly different sites differed in methylation to only a small degree in absolute terms (≤0.2). Changes in these sites are consistent with the aforementioned “epigenetic drift” where shifts in methylation affect only a random subset of cells. Since DNA methylation values for a single site on a single chromosome are either 0 or 1, and given that DNA methylation is stochastic, values less than 0.1 indicate that no more than 10 % of the cell population have acquired a methyl group at a given CpG location. Nonetheless, our data show the expected asymmetry in methylation changes with age, indicating a propensity to accumulate DNA methylation with the transition from fetal life to adulthood.

A high proportion of DNA methylation changes, even those in gene regions expected to exert an effect on transcription, were unassociated with changes in gene expression. The methylation pattern in fetal liver was consistent with a relationship between hypomethylation and increased gene expression. However, the fetal methylation pattern for these fetal overexpressed genes was similar to the pattern for genes expressed at similar levels in fetus and adult. In contrast, genes overexpressed in the adult tissue showed patterns that differed from those seen for similarly expressed genes. Hypermethylation in the fetus was associated with overexpression in the adult. However, very few genes were hypomethylated in the adult. Rather, there appeared to be a relationship between higher gene expression in the adult and intermediate adult methylation. This is consistent with the conclusions of Bestor et al. [31] who posited that the accumulation of DNA methylation later in life may not be a regulator of gene expression.

This is in contrast to hypermethylation in fetal liver. The highly methylated CpGs in fetal liver may represent a set of actively methylated sites that are independent of X-inactivation or transposon silencing. Given the relationship between fetal hypermethylation of TSS/Ex1 CpGs and higher gene expression in the adult, we speculate that these sites may contribute to the induction of genes associated with functional differentiation in the adult. This is supported by the preponderance of genes encoding cytochrome P450 enzymes among those that underwent a reduction in methylation from fetus to adult. Such a relationship would be similar to that reported by Kacevska et al. for *CYPA4* [32]. These authors found hypermethylation of key upstream regulatory sites in this gene in fetal relative to adult human liver.

As in our own analyses, Bonder et al. [33] analyzed the transcriptomes and DNA methylomes of human fetal and adult liver. Fetal samples were acquired at gestational week 8–12, a substantial difference from our own study. In general, these authors’ findings were consistent with many of our observations. Methylation within promoter regions tended to be associated with negative regulation with gene expression, as opposed to methylation within gene bodies. The authors attributed this to the ability of methylation to inhibit transcription initiation but not transcription elongation [22]. In contrast to our results, these authors found that only 8 % of CpGs showed a significant difference in beta of >0.2 comparing the fetal and adult samples. This may have related to the gestational age span of the fetal samples. However, like our analyses, they found a slight hypomethylation preponderance of CpGs in fetal liver. Consistent with our findings, they saw that CpGs hypomethylated in fetal liver were enriched among genes encoding components of steroid and lipid metabolic pathways.

An additional focus of our analyses was the relationship between localization of methylation within distinct gene regions and effects on gene expression. It has been noted previously that methylation within promoter regions is low relative to the higher rates seen within the gene body, the latter being primarily within exons [5]. Brenet et al. [25], studying human cell lines, noted that methylation within a gene’s first exon is more tightly linked to silencing of transcription than is methylation upstream of the transcription start site. Filtering of our methylation data for location and a significant fetal-to-adult reduction in methylation at specific sites was most effective in identifying candidate regulatory methylation sites. However, even those sites identified by the most stringent predictor (a significant fetal-to-adult decrease of at least 0.5 in a TSS/Ex1 site) were unassociated with significant changes in gene expression two thirds of the time. Furthermore, promoter regions and exon 1 sites were not distinguishable from one another in their relationship to gene expression. It is evident from our data that epigenetic control of gene expression via DNA methylation is not only extremely complex, but that the nature of its relationship to gene expression is, as yet, unpredictable. Our data indicate that in human liver tissue a large percentage of statistically significant changes in DNA methylation are unrelated to the regulation of gene expression during the transition from fetal to adult life.

Multiple regulatory mechanisms are likely to account for the regulation of gene expression during liver development. Regulation of gene expression is exceedingly complex and involves the interplay between chromatin structure and expression and activity of transcription factors. Specific histone modifications are present in the mouse endoderm and have been linked to lineage specification of liver and pancreas [34]. Dynamic changes in histone posttranslational modifications are also involved in the developmental regulation of Cyp3a genes in mouse liver [35]. Several studies have also shown changes in the profile of miRNAs during liver development with a subset being specifically expressed in fetal liver suggesting an important role in the regulation of gene expression [36–38].

However, we also identified a subset of genes that are targeted for DNA methylation during fetal development when the expression of these genes is down-regulated. The expression of these genes in adulthood correlates with intermediate methylation. This is consistent with the demethylation of one copy of the gene during active gene transcription. It remains to be determined if these genes represent reversible gene silencing and, if they do not, why these upstream CpGs are targeted for methylation during fetal development when upstream CpGs are preferentially unmethylated in fetal tissue. Finally, our data indicate that methylation, within promoter regions or first exons in particular, has a regulatory influence on genes that are overexpressed in adult relative to fetal liver. The converse was not the case; a transition from hypomethylation in the fetus to hypermethylation in the adult was not associated with altered gene expression. These results indicate a fundamentally different role for methylation in the regulation of genes that are inhibited versus induced during the transition from fetal to adult life.

Our results indicate that in a physiological setting there exists a relationship between DNA methylation and gene expression among genes that are induced beyond mid-gestation. The corollary relationship between genes overexpressed in the fetus and increased methylation in the adult is not similarly supported by our data. We therefore conclude that there is a fundamentally different relationship between DNA methylation and gene expression among genes that are hypermethylated in the fetus versus those that contribute to the trend toward increasing methylation in the adult.

## Conclusions

Nearly half of the CpGs in human liver show a significant difference in methylation comparing mid-gestation fetal and adult samples. While most methylation events are not associated with altered gene expression, sites within promoter regions or first exons that show a transition from hypermethylation in the fetus to hypomethylation or intermediate methylation in the adult are associated with inverse changes in gene expression. In contrast, increases in methylation going from fetal to adult appear to neither regulate nor reflect fetal-to-adult decreases in expression. These findings indicate fundamentally different roles for and/or regulation of DNA methylation in human fetal and adult liver.

## Methods

### Liver tissue procurement and processing

Human fetal liver was obtained from spontaneous male fetal stillbirths, gestational weeks 20–22, delivered at Women and Infants Hospital (Providence, RI). Macerated fetuses, presumed to have a long period between fetal demise and delivery, and fetuses with visible congenital abnormalities or those delivered by elective or medical abortion were excluded. Details of fetal examination were described by De Paepe et al. [39]. Once obtained, the fetal liver was split and a portion of the liver immediately flash frozen in liquid nitrogen and stored at −80 °C until use. The other portion was fixed in formalin and processed for hematoxylin and eosin staining. The tissue procurement protocol was approved by the Women and Infants Institutional Review Board and fully informed written parental consent was obtained according to the approved protocol.

Snap frozen, normal adult human liver from males aged 55–62 years was obtained through the Liver Tissue Cell Distribution System (Pittsburgh, PA), which is funded by NIH Contract # HHSN276201200017C. Specimens were stored at −80 °C until use.

### Gene expression analysis

DNA and RNA were isolated from triplicate human fetal and adult livers using the AllPrep DNA/RNA mini Kit (Qiagen, Redwood City, CA). RNA quality was assessed using the Agilent 2100 Bioanalyzer (Santa Clara, CA). RNA was of high quality with all samples giving a RNA integrity number (RIN) between 9.5 to 9.7. RNA was analyzed using Affymetrix Genechip Human Gene ST 1.0 arrays (Affymetrix, Santa Clara CA) by Brown University’s Genomics Core.

The Affymetrix CEL files were imported into R and normalized using the RMA algorithm and then each transcript was associated with standard gene symbols (packages oligo [http://www.ncbi.nlm.nih.gov/pubmed/20688976] and hugene10sttranscriptcluster.db [40]). Gene expression and statistical significance of differences between fetal and adult samples were calculated using the limma package [41]. Microarrays were analyzed using Gene Set Enrichment Analysis to identify functional gene sets that were enriched in experimental groups for the full spectrum of genes detected in the array [21]. For Ingenuity Pathway Analysis (IPA®; Qiagen, www.qiagen.com/ingenuity), genes whose expression differed significantly (FDR *q* < 0.05) between the fetal and adult groups by >5-fold were compared to 5 equally sized groups of control genes that were randomly selected from the list of genes that showed a fold-change <1.1 [42]. IPA results were considered significant when the *p*-values were below the lowest *p*-value for the 5 control gene sets.

### DNA methylation analysis

DNA quality was assessed using a Nanodrop ND 1000 (Thermo Scientific, Wilmington, DE) and gel electrophoresis. All samples contained high molecular weight genomic DNA with 260/280 greater than 1.8 and 260/230 greater than 1. DNA (3 μg) was sent to the W.M. Keck Biotechnology Resource Laboratory at Yale University for methylation profiling using the Illumina Infinium® HumanMethylation450 BeadChip (Illumina, San Diego, CA). This array includes over 489,000 methylation sites across the entire human genome. The chip was designed to cover 99 % of RefSeq genes and different locations within the promoter region, 5’ and 3’ UTRs, and the body of the gene. The gene body sites explicitly include the first exon. Samples were processed for hybridization according to the manufacturer’s instructions.

Raw DNA methylation array data files were imported into the R statistical package [43]. The probesets were quality filtered for adjusted detection *p*-values <0.05, batch corrected with functional normalization [44], and then normalized across the two probe types using beta mixture quantile normalization (BMIQ) [45], all implemented in the watermelon package [46]. Our final probeset included 482,757 methylation sites along the genome. We then linked the UCSC Genome Browser annotation (version hg19 of the human reference genome, http://genome.ucsc.edu) to each of the CpG sites on the array [47]. Based on the UCSC chromosome annotation, we filtered out DNA methylation from the X and Y chromosomes. The resulting methylation values follow a beta distribution ranging from 0 if potential sites in the sample are unmethylated at a locus to 1 if all are methylated. Beta values between 0 and 1 reflect the proportion sites methylated at a given locus. Fetal:adult differences are referred to as delta beta values.

We used surrogate variable analysis [48] to remove possible confounding latent variables associated with donor characteristics followed by linear modeling and empirical Bayes moderation [49] and a Benjamini-Hochberg false discovery rate correction [50] on the logit-transformed beta methylation values of the adult versus fetal samples [48].

### Availability of supporting data

The data sets supporting the results of this article are available in the Gene Expression Omnibus (GEO) repository, GSE69852 for gene expression data and GSE69713 for methylation data.

## Abbreviations

GSEA: Gene set enrichment analysis
FDR: False discovery rate
IPA: Ingenuity pathway analysis
dβ: delta-beta
TSS: Transcription start site
Ex1: exon 1
GB: Gene body
UTR: Untranslated region
GEO: Gene expression omnibus
